# From Surviving to Living (on): A Grounded Theory Study on Coping in People with Pancreatic Cancer

**DOI:** 10.1177/23743735231215605

**Published:** 2023-11-20

**Authors:** Patrick Ristau, Claudia Oetting-Roß, Andreas Büscher

**Affiliations:** 1 Faculty of Health, School of Nursing Science, 12263 Witten/Herdecke University, Witten, Germany; 2 Institute for Social Medicine and Epidemiology, Nursing Research Unit, 9191 University of Lübeck, Lübeck, Germany; 3 Münster Department of Health, FH Münster, University of Applied Sciences, Münster, Germany; 4 Faculty of Business Management and Social Sciences, 38924 Osnabrück University of Applied Sciences, Osnabrück, Germany

**Keywords:** patient experience, pancreatic carcinoma, adaptation, psychological, patient-centered care, psycho-oncology, trajectory

## Abstract

Little research has been conducted on the experience of pancreatic cancer from a patient's perspective. Several factors suggest that trajectory models of chronic illness or other cancers cannot be applied to pancreatic cancer. Within this grounded theory study, 26 problem-centred interviews were conducted with people with pancreatic cancer from Germany. A cancer-specific trajectory model was developed, depicting both curative and palliative courses. Two successive phases form the core: Immediately after diagnosis, there is an acute phase in which patients focus on mere survival, attempt to overcome the short-term consequences of pancreatic cancer and search for information. This initial phase is followed by a circular phase of living on with pancreatic cancer, characterized by adaptation to the long-term consequences of the disease and a repeated experience of fear of recurrence or progression and threat in the context of follow-up examinations. Understanding disease trajectories from a patient's perspective enables health professionals better to understand patients’ needs, concerns, and fears and better support them in coping. Trial registration: German Clinical Trials Register, DRKS00020251, 13.01.2020.

## Key Findings

1. A pancreatic cancer-specific and comprehensive trajectory model was developed from the experience and perspectives of both curatively and palliatively treated patients.
2. An initial phase following diagnosis, characterized by the pure will to survive, transitions into a second phase of living with pancreatic cancer.
3. In the first phase, searching for information and coping with acute disease-related challenges depletes most patients’ capacities.
4. Since everything else is subordinated to survival, informed consent to treatment options and weighing up the chances, risks, and consequences do not usually seem realistic for people with pancreatic cancer.
5. Living on with pancreatic cancer is characterized by the best possible adaptation to the long-term consequences of the disease and a recurring threat and fear of recurrence or progression in the context of follow-up examinations.

## Introduction

Pancreatic ductal adenocarcinoma—which accounts for 9 out of 10 of all pancreatic malignancies—is an early metastatic malignancy characterized by three factors^1,2^: (1) diagnosis usually at an advanced stage, (2) rapid progression, (3) and poor prognosis. Only early, localized stages allow for the best possibility of curative therapy—complete tumor removal—while distant metastases lead to palliative therapy.^1,3^ In the recent decade, advances in surgical techniques and new chemotherapies slightly improved median survival.^1,4^ However, survival rates remain poor compared to other cancer entities, and early detection tests are still lacking.^1,4,5^

People experience the diagnosis of pancreatic cancer as a life-changing event and a sudden and acute life threat that triggers existential fears.^6^ Pancreatic cancer leads to numerous physical, psychological, and social consequences. Accordingly, people face multiple changes in their quality of life and coping tasks. Previous research questions whether concepts such as shared decision-making can be applied in the context of therapy decisions.^6^

People will indefinitely continue to live with their disease after the initial diagnosis and therapy of pancreatic cancer—ranging from a few months to several years. Illness trajectories^7,8^ illustrate this only in general but not in disease-specific terms. There is no comprehensive model of how people with pancreatic cancer cope with disease-related challenges. Whether and how they integrate their illness and its consequences into their everyday lives remains unclear. Professional associations’ calls to action—which also emphasize the patient perspective—focus solely on optimizing treatment and outcomes.^9^ However, there are several advantages to knowing the perspective of those affected: Their view allows for understanding challenges, needs, and approaches to cope with them first-hand and subsequently enables professionals to support them most effectively and comprehensively.

Our study aims to close this gap in research by developing a pancreatic cancer-specific trajectory model—drawn from patients’ perspectives. Their views provide the basis for a tangible illustration of the coping processes and developments over time.

## Methodology and Methods

This qualitative study follows Strauss and Corbin's approach to grounded theory.^10,11^ It was preregistered (German Clinical Trials Register, DRKS00020251, 13.01.2020) and approved by the Ethics Committee of Witten/Herdecke University (141/2019). In presenting the results, we follow the Standards for Reporting Qualitative Research^12^ and the Consolidated criteria for Reporting Qualitative Research^13^ (see Appendices 1 and 2).

### Field Access and Recruitment

A nationwide self-help group provided access to the field by disseminating the call for participation. Adults with pancreatic adenocarcinoma were recruited regardless of their disease stage or treatment goals. Participants gave verbal and written consent and declared ongoing consent on the interview day.^14^

### Sampling and Sample

The initial sample consisted of the first four people willing to be interviewed, followed by open, purposive, theoretical, and discriminatory sampling. Approximately 30 people responded to the call for participation, resulting in 26 interviews (Table 1). In parallel, codes and categories were derived and developed from the material and finally synthesized in our model.^10,11,15^ To our regret, two people passed away before their interview.

**Table 1.** Characteristics and Descriptions of the Sample and the Interviews.

Characteristics	Description
Sample size	*N* = 26
Sex (female/male)	16/10
Therapy (curatively intended/palliative)	61.5%/38.5%
Mean age (*Mdn, Range, SD*)	68.4 years (70.5; 52-82; 8.9)
Mean time since diagnosis (*Mdn, Range, SD*)	2.9 years (2; 0-19^a^; 3.8)
Curatively intended therapy strategy (*Mdn, Range, SD*)	3.4 years (2; 0-19; 4.5)
Palliative therapy strategy (*Mdn, Range, SD*)	1.7 years (1; 0-5; 1.8)
Face-to-face/online/telephone interviews	11/14/1
Average interview duration (*Mdn*, *Range, SD*)	01:16 h (01:10; 00:37-03:08; 00:30)

^a^ The sample included one long-term survivor (19 years since diagnosis and curative therapy). Without this person, the range would have been 0-7 years.

### Data Collection and Analysis

An alternating and complementary data collection and analysis characterizes the grounded theory research process.^10,11,15^ For this purpose, problem-centred and guideline-based interviews were conducted face-to-face, on the phone or online. Subsequently, the audio recordings were transcribed verbatim and anonymized.^16–19^ The interview guide is presented in Appendix 3. Memos of impressions during the interview, specific thoughts and ideas supplemented these qualitative data, and theory links were recorded immediately after each interview.^10,11^

**Figure 1.**
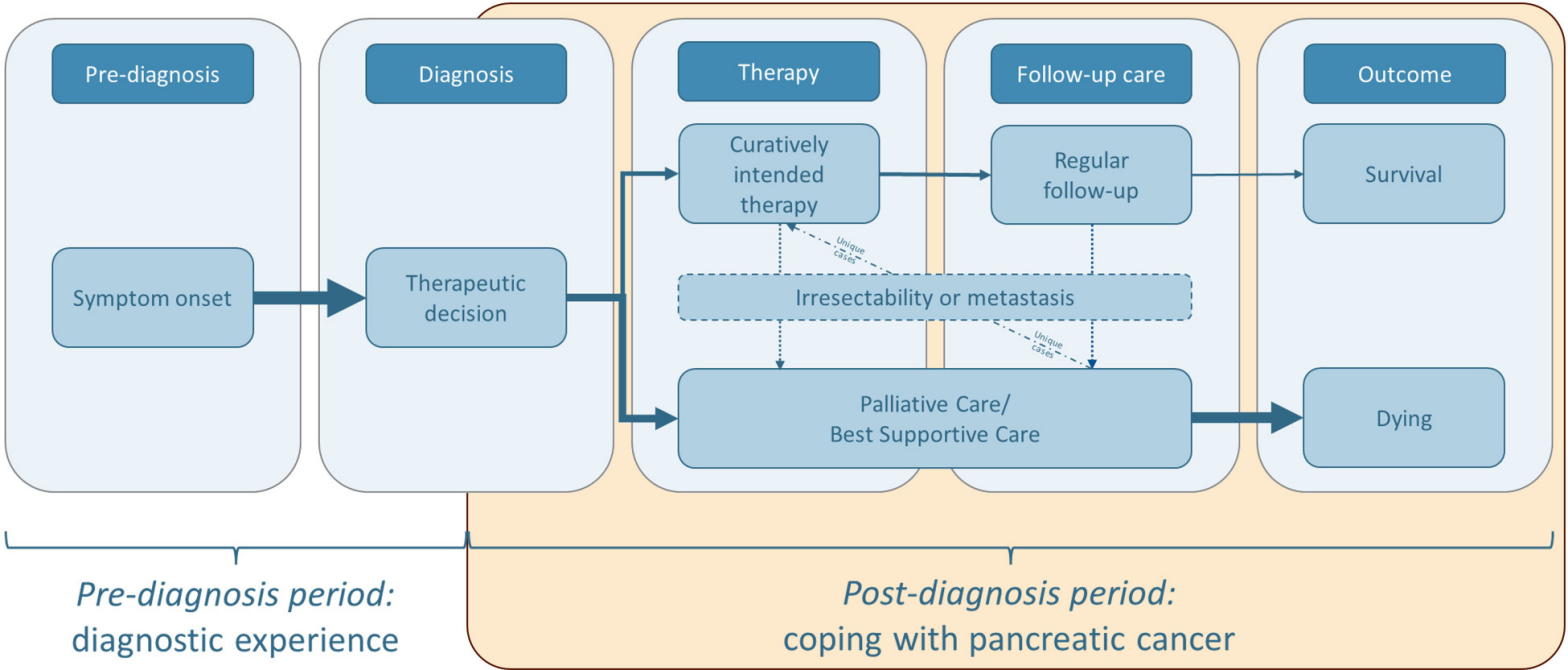
The phase-wise trajectory of pancreatic cancer derived from the data provided the framework for our analysis of coping with it.

Three interviews were initially open-coded.^10,11^ Strauss and Corbin's coding paradigm was used for the axial coding of these and all other transcripts using MAXQDA 2022. Furthermore, the categories were dimensionalized (eg, *durations* fast—slow or *frequencies* often—seldom). Here it became apparent that all interviewees clearly distinguished between *prediagnosis* and *postdiagnosis periods* and the related *diagnostic experience* respectively *coping with pancreatic cancer* (Figure 1). This phenomenon has been described in the literature in the context of chronic or life-limiting illnesses.^8,20,21^ Given this fact, we decided to analyze the two time periods and the phenomena described separately. This publication refers to the *postdiagnosis period,* which begins immediately after the diagnosis.

Selective coding was used to examine the categories for an overarching structure. These relationships were subsequently validated against the data and supplemented with additional material.^10,11^ Selective coding enabled us to relate the core categories to other subcategories to achieve a more abstract level of analysis. Finally, three interviews were conducted to validate the developed model.

## Results

**Figure 2.**
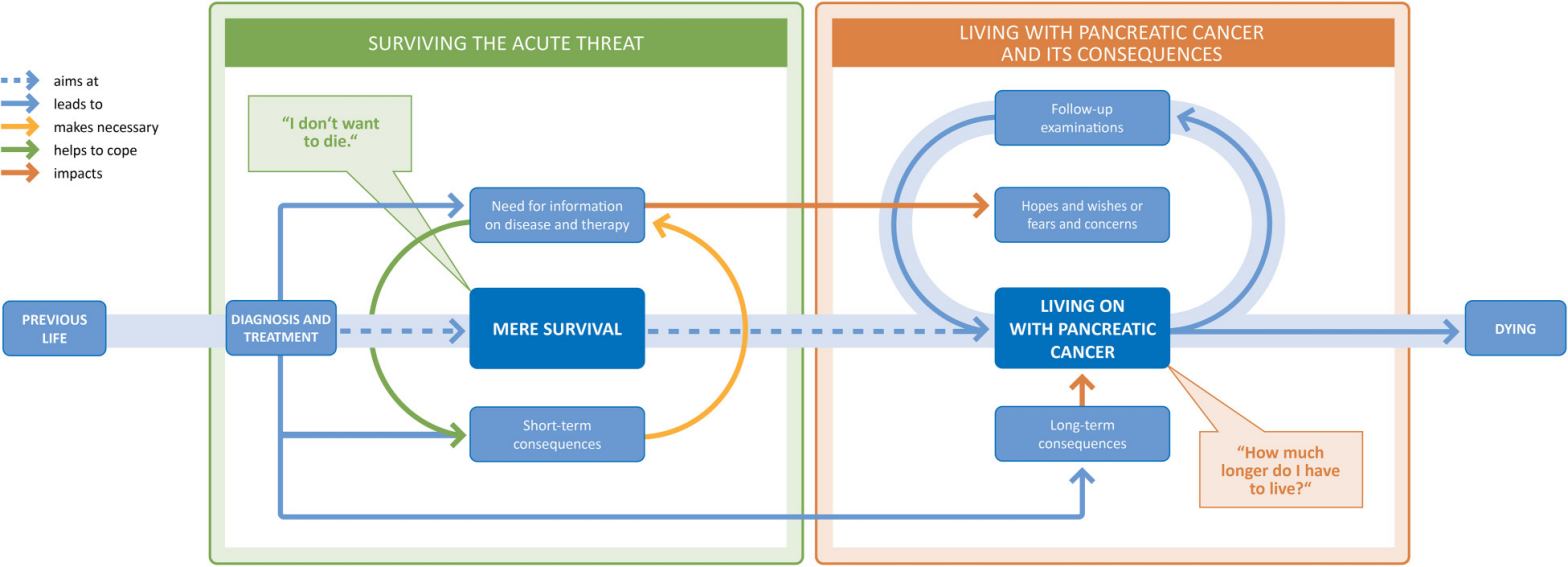
Trajectory model of coping with pancreatic cancer.

Individuals with pancreatic cancer experience their disease in two consecutively occurring phases—regardless of any subsequent treatment strategy and whether it is curable or incurable. During the first phase, which immediately follows the diagnosis, the focus is on mere survival. The second phase is about living on with and adjusting to the consequences of pancreatic cancer and its therapy. Figure 2 shows our trajectory model for coping with pancreatic cancer. Its sections, components, links, and interrelationships are explained below.

### Surviving the Acute Threat

Given the diagnosis of pancreatic carcinoma, the first phase focuses on simply surviving the acute situation, borne by the uppermost goal of not wanting to die—regardless of whether it is a potentially curable tumor. Both diagnosis and therapy lead to a great need for information in those affected. In summary, hope and desire for survival are fundamental in this acute phase after diagnosis, regardless of the prognosis.

### Need for Information on Disease and Therapy

Being diagnosed with pancreatic cancer raises many questions. Many cannot understand the consequences immediately in those acute situations and need more information about their illness and prognosis. Furthermore, they search for reports on the experiences of other sufferers—especially those who have survived their disease despite a poor prognosis. The internet is their primary source of information. GPs and specialists are sometimes consulted; self-help groups play a secondary role. Comprehensive and understandable information can help patients cope with pancreatic cancer's short-term consequences.

Simultaneously, patients usually learn about the poor prognosis of pancreatic cancer through their research or previous knowledge, which leads to significant worries and existential fears about the future. At the same time, this information and anxieties also influence how hopes, wishes, fears, or concerns are dealt with later. Another noteworthy aspect of information research, especially in palliative situations, is that those affected actively look for ways to fight their disease.

### Short-Term Consequences of Pancreatic Cancer

At the same time, therapy initiation is usually rapid while patients still lack comprehensive information: Usually, surgical therapy takes place within a few days without patients seeking a second opinion. Thus, it is often up to the physician to decide on the usefulness of treatment options for their patients, who, in turn, usually do not question the decision. Because of the severity of the surgical intervention, patients immediately experience its consequences: Besides acute complications such as pain or an inability to eat, many patients presently have difficult-to-control diabetes and massive digestive problems. Teaching how to take pancreatic enzymes and manage pancreoprivic diabetes is prone to error in many hospitals due to a lack of experience and knowledge of the medical and nursing staff, as reported by the study participants. Generally, in this acute situation, people with pancreatic cancer are forced to face their health problems. They react by gathering information as described above, while they are also forced to endure the symptoms and side effects and feel correspondingly helpless.

Neoadjuvant chemotherapies are understood as a chance for and prerequisite for a possible cure. In contrast, the chemotherapies following the surgery are experienced as physically and psychologically incredibly stressful and full of side effects, sometimes leading to early discontinuation. In some cases, participants can integrate prolonged chemotherapies into their daily lives. For those weakened by the surgery, adjuvant chemotherapy is initially not an option. This circumstance seems to raise a question of guilt in the event of a later relapse.

Rehabilitation programs are attributed to comparatively low effects from a patient's point of view, as they often fail to meet individual needs.

### Overarching Consequences and Transition to Living With Pancreatic Cancer

In general, a high symptom burden after surgery increases the suffering of those affected to such an extent that, given the significant loss of quality of life, they ask themselves whether the surgery had been the right thing to do. Conversely, patients with advanced, noncurable diseases often show few or no symptoms and do not experience any physical restrictions in their quality of life during this phase.

The transition to living on with pancreatic cancer can happen at different points: After hospitalization, after chemotherapy, or even during—depending on when and how it is possible for those affected to integrate their illness and its consequences into their everyday lives. First, this requires a stable course of the disease so that patients can adapt to the new life conditions resulting from their state of health.

### Living With Pancreatic Cancer and Its Consequences

In the second phase of the model, people with pancreatic cancer arrange their lives around their disease's longer-term consequences and developments. The question about the remaining time of life with simultaneous awareness of one's finiteness manifests itself in a field of tension of crucial importance: It spans hopes and wishes on the one hand and fears and concerns on the other and influences everyday life to varying degrees. The whole life of people with pancreatic cancer is repeatedly endangered by follow-up examinations, which they perceive as an acute threat, as each carries the possibility of discovering that the cancer has progressed or recurred.

### Long-Term Consequences of Pancreatic Cancer

Long-term consequences of pancreatic cancer cause various changes, which influence life in many ways. These are integrated as well as possible into living with pancreatic cancer. Changes unfold on physical, psychological, and social levels, including positive and negative consequences, which can arise in various combinations and partly be mutually dependent. Table 2 provides an overview.

**Table 2.** Possible Physical, Psychological, and Social Consequences in the Course of Pancreatic Cancer.

	Positive consequences	Negative consequences
Physical level	• Altered body awareness • Weight gain posttherapy • Ability to eat again • Few symptoms (if no surgery) • Increased awareness of sensory impressions	• Fatigue • Lack of strength • Polyneuropathy • Digestive problems • Hard to control blood glucose levels
Psychological level	• Shifting priorities • Increased resilience • Increases self-confidence • Acceptance of the overall situation	• Fixation on negative consequences • Not accepting the overall situation • Reduced resilience • Reduced self-esteem • Depression • Panic attacks
Social level	• Intensification of the relationship between spouses/partners • Receiving and accepting social support • Acceptance of new roles	• Blaming oneself • Loss of social roles and restriction to the role of the sick person • Social pressure and sense of obligation towards family (and friends) • Withdrawal from the social environment/family • Inability to work or retirement • Financial worries

### Living on with Pancreatic Cancer

People with pancreatic cancer shape their everyday lives around the long-term consequences of the disease and try to integrate these into their daily lives as much as possible.

This is possible to a great extent in cases where there are hardly any physical symptoms—as can be the case, for example, with people receiving palliative treatment other than chemotherapy. They can engage in their daily activities and follow a regular daily structure. Completing tasks and goals such as shopping or gardening is essential for self-awareness, giving the affected person strength and confidence. In addition, pancreatic cancer can also play a secondary role alongside other diseases, some of which are age-related. In summary, those affected with a low symptom burden describe a quality of life subjectively perceived as good, regardless of the individual prognosis.

On the other hand, the entire life of those affected may change, for example, if they need to retire from work due to their symptom burden. Additionally, severe symptoms such as persistent and difficult-to-control digestive problems, fatigue or lack of strength may force patients to withdraw socially. A reduced resilience, depression, or panic attacks can affect life negatively. Some even regret undergoing surgery because of the high symptom burden afterwards. In addition, patients may not want to be a burden to others. A supportive environment can partially compensate for some of the effects described above. For example, some general practitioners coordinate further treatment and issue prescriptions as needed.

The close social environment—especially family or spouses—can have both a supportive effect and an opposite effect: Participants regularly reported that either couple relationships intensify and the partners try to overcome this crisis together, or they feel misunderstood by their closest relatives. The latter can especially be the case when patients have accepted their palliative state but relatives are not (yet) able to do so. In addition, the appearance of the person affected—especially in palliative cases—does not allow any conclusions to be drawn about the actual state of health: The social environment often misinterprets a good appearance. Because of this discrepancy, attempts at encouragement and help may be rejected. Overall, however, the relationship with relatives seems to play a minor role; the central issue is how the person affected deals with their illness of pancreatic cancer.

People living with pancreatic cancer develop an awareness of their disease and their finiteness during this phase of their disease. These thoughts can lead to melancholy—especially if they are accompanied by feelings of having missed out in the past—but in many cases, they recede into the background. Instead, patients give way to an “it comes as it comes,” which can be interpreted as a sign of acceptance of illness and fate.

### The Tension Between Hopes and Wishes or Fears and Concerns

A tension between hopes and wishes or fears and concerns shapes an individual's life: People with pancreatic cancer compare themselves—especially in self-help groups—with other patients. They learn about cases in which others have lived for years, which gives them strength and courage. However, they are also confronted with the deaths of others. They transfer positive aspects from other cases to themselves but reject negative aspects by arguing that everyone is different and, therefore, no conclusions about their own disease could be drawn.

In many cases, the individual severity of the disease, derived from medical characteristics, and the individual hope of those affected appear as two sides of the same coin. Fears about the future due to knowledge about the disease are contrasted with the hope of living on (as long as possible) or the hope for new therapies. Patients plan their short-term future while, at the same time, they hope to have an exceptional outcome. Furthermore, wishes for a possibly self-determined and suffering-free death are expressed.

### Follow-up Examinations

Participants reported that they regularly feel a lot of concern and anxiety before follow-up examinations—regardless of their therapy goals and even years after the diagnosis: Each time a cancer recurrence or disease progression might be diagnosed. Therefore, before each follow-up, they feel their lives are acutely and existentially threatened. This process repeats itself: after a check-up, people either go on with their lives or adapt to changes—until the next check-up. In summary, living with pancreatic cancer is experienced as fragile because of the recurring threat, regardless of how patients have otherwise arranged their lives around their disease.

## Discussion

Within this study, we developed a cancer-specific trajectory model for experiencing pancreatic cancer and dealing with its challenges from a patient's perspective. Irrespective of the patient's treatment strategy or prognosis, our model provides insight into how people respond to the challenges of having pancreatic cancer. It integrates the acute phase immediately after diagnosis, focusing on survival, and the second phase following the acute therapies and treatments, when the focus shifts to living on with pancreatic cancer and its consequences, thereby enabling a holistic view of the experience of illness.

So far, a pancreatic cancer-specific trajectory model has been lacking to understand the effects on patients. Although pancreatic cancer—like many other cancers and chronic diseases—marks a disruptive biographical event,^22,23^ it distinguishes from many other cancer entities as described above. Since it is questionable whether people with pancreatic cancer ever reach a chronic stage without the threat of cancer recurrence or progression—our results show that pancreatic cancer is circular precisely around this phenomenon. Accordingly, neither linear progression models nor chronic disease trajectory models are transferable. However, this may not be true for people close to death.^24^

Our results significantly extend the findings of Schildmann et al.^25^ on the perceptions and views on information and treatment decision-making towards a pancreatic cancer-specific trajectory framework and, in particular, clarify the overarching motivations of action in the acute and longer-term phases of living with pancreatic cancer. Furthermore, they confirm and extend the findings of Wancata et al.^26^ and Taylor et al.^27^ on patients with pancreatic cancer who underwent chemotherapy and surgical resection. Our model additionally incorporates the perspective of those patients with palliative treatment and offers a therapy goal-independent trajectory model.

Furthermore, the relationship of trust between doctor and patient is significant, especially in the acute phase, whereby the physician's sovereignty of information and thus decision-making in the acute phase, but also their responsibility, should be mentioned in particular.^6,25,28,29^ Patients express that they decide for or against therapy and consider therapy as the only option.^26,28^ Against this backdrop, our study suggests that informed consent may be impossible to obtain in the acute phase due to the sheer will to survive; some patients even regret surgery later.^30^

At the same time, our study answers why people with advanced pancreatic cancer do not give up hope of (continuing) life even in medically discouraging situations while adapting to new living conditions yet constantly threatened by the fear of cancer recurrence or progression described in the literature.^25,27,31,32^ The patients’ realistic understanding of the life-limiting prognosis described by Wancata et al.^26^ was also found clearly in our study but took a back seat to the question of general time remaining and the associated recurrent uncertainty.

Consequently, the question of whether living with pancreatic cancer is classic survivorship and whether this term reflects patients’ realities arises. This should be questioned regarding the prognosis, the trajectories, and the ongoing adjustment of life to the consequences of pancreatic cancer described in this study. Therefore, less loaded terms such as “aliver” or “thriver” may be more appropriate in the context of pancreatic cancer.

## Strengths and Limitations

Our study includes both curative and palliative pancreatic cancer patients. The methodological approach and sampling strategies were appropriate. Of note is that this study took place against the backdrop of the German healthcare system, and participants were recruited through a self-help group. The results are not easily transferable to patients in other healthcare systems or socio-cultural backgrounds. In addition, people open to self-help groups may have additional resources to cope with a serious, life-limiting illness.^33,34^

## Conclusion

Our research provides—for the first time—a unique view of the trajectories of pancreatic cancer from a patient's perspective by focusing on their experience. Thus, this work goes beyond articulating unmet needs^9^: The *genuine* patient perspective—considering differences between the perceptions of health professionals and their patients^35^—can help to better understand patients’ needs, concerns and fears and better support them in coping.

## Supplemental Material

sj-docx-2-jpx-10.1177_23743735231215605 - Supplemental material for From Surviving to Living (on): A Grounded Theory Study on Coping in People with Pancreatic Cancer Click here for additional data file. Supplemental material, sj-docx-2-jpx-10.1177_23743735231215605 for From Surviving to Living (on): A Grounded Theory Study on Coping in People with Pancreatic Cancer by Patrick Ristau, Claudia Oetting-Roß and Andreas Büscher in Journal of Patient Experience

sj-docx-3-jpx-10.1177_23743735231215605 - Supplemental material for From Surviving to Living (on): A Grounded Theory Study on Coping in People with Pancreatic Cancer Click here for additional data file. Supplemental material, sj-docx-3-jpx-10.1177_23743735231215605 for From Surviving to Living (on): A Grounded Theory Study on Coping in People with Pancreatic Cancer by Patrick Ristau, Claudia Oetting-Roß and Andreas Büscher in Journal of Patient Experience

sj-docx-4-jpx-10.1177_23743735231215605 - Supplemental material for From Surviving to Living (on): A Grounded Theory Study on Coping in People with Pancreatic Cancer Click here for additional data file. Supplemental material, sj-docx-4-jpx-10.1177_23743735231215605 for From Surviving to Living (on): A Grounded Theory Study on Coping in People with Pancreatic Cancer by Patrick Ristau, Claudia Oetting-Roß and Andreas Büscher in Journal of Patient Experience
