# Supplementary material for: From Surviving to Living (on): A Grounded Theory Study on Coping in People with Pancreatic Cancer
Source: J Patient Exp. 2023 Nov 20;10:23743735231215605. doi: 10.1177/23743735231215605 (PMC10750439; doi:10.1177/23743735231215605)
Supplement: sj-docx-3-jpx-10.1177_23743735231215605 - Supplemental material for From Surviving to Living (on): A Grounded Theory Study on Coping in People with Pancreatic Cancer [file sj-docx-3-jpx-10.1177_23743735231215605.docx]

| **Appendix 2: COnsolidated criteria for REporting Qualitative studies (COREQ): 32-item checklist** | | | |
| --- | --- | --- | --- |
|  | | | |
| Adapted from: Tong, A., Sainsbury, P., & Craig, J. (2007). Consolidated criteria for reporting qualitative research (COREQ): a 32-item checklist for interviews and focus groups. *International journal for quality in health care : journal of the International Society for Quality in Health Care, 19*(6), 349–357. [https://doi.org/10.1093/intqhc/ mzm042](https://doi.org/10.1093/intqhc/mzm042) | | | |
|  |  |  |  |
| **No** | **Item** | **Guide questions/description** | **Answer/explanation** |
| **Domain 1: Research team and reflexivity** | | | |
| Personal Characteristics | | | |
| 1. | Interviewer/facilitator | Which author/s conducted the interview or focus group? | PR |
| 2. | Credentials | What were the researcher's credentials? *E.g. PhD, MD* | See title page |
| 3. | Occupation | What was their occupation at the time of the study? | See title page |
| 4. | Gender | Was the researcher male or female? | Male |
| 5. | Experience and training | What experience or training did the researcher have? | See title page |
| Relationship with participants | | | |
| 6. | Relationship established | Was a relationship established prior to study commencement? | No |
| 7. | Participant knowledge of the interviewer | What did the participants know about the researcher? e*.g. personal goals, reasons for doing the research* | Reasons for doing the research |
| 8. | Interviewer characteristics | What characteristics were reported about the interviewer/facilitator? e.g. *Bias, assumptions, reasons and interests in the research topic* | Doctoral student status |
| **Domain 2: study design** | | | |
| Theoretical framework | | | |
| 9. | Methodological orientation and Theory | What methodological orientation was stated to underpin the study? *e.g. grounded theory, discourse analysis, ethnography, phenomenology, content analysis* | Grounded theory following Strauss and Corbin |
| Participant selection | | | |
| 10. | Sampling | How were participants selected? *e.g. purposive, convenience, consecutive, snowball* | The initial sample consisted of the first four people willing to be interviewed, followed by open, purposive, theoretical, and discriminatory sampling. |
| 11. | Method of approach | How were participants approached? e*.g. face-to-face, telephone, mail, email* | A nationwide self-help group provided access to the field by disseminating the call for participation. |
| 12. | Sample size | How many participants were in the study? | 26 |
| 13. | Non-participation | How many people refused to participate or dropped out? Reasons? | 2 passed away before an interview could be scheduled |
| Setting | | | |
| 14. | Setting of data collection | Where was the data collected? e*.g. home, clinic, workplace* | Face-to-face at participants‘ homes, online, via telephone |
| 15. | Presence of non-participants | Was anyone else present besides the participants and researchers? | No |
| 16. | Description of sample | What are the important characteristics of the sample? *e.g. demographic data, date* | See Table 1 |
| Data collection | | | |
| 17. | Interview guide | Were questions, prompts, guides provided by the authors? Was it pilot tested? | No, not to participants/yes |
| 18. | Repeat interviews | Were repeat interviews carried out? If yes, how many? | No |
| 19. | Audio/visual recording | Did the research use audio or visual recording to collect the data? | Audio recording |
| 20. | Field notes | Were field notes made during and/or after the interview or focus group? | Yes |
| 21. | Duration | What was the duration of the interviews or focus group? | See Table 1 |
| 22. | Data saturation | Was data saturation discussed? | Yes |
| 23. | Transcripts returned | Were transcripts returned to participants for comment and/or correction? | No |
| **Domain 3: analysis and findings**z | | | |
| Data analysis | | | |
| 24. | Number of data coders | How many data coders coded the data? | One main coder, supplemented by interpetation partnerships |
| 25. | Description of the coding tree | Did authors provide a description of the coding tree? | N/A |
| 26. | Derivation of themes | Were themes identified in advance or derived from the data? | From the data |
| 27. | Software | What software, if applicable, was used to manage the data? | MAXQDA 2022 |
| 28. | Participant checking | Did participants provide feedback on the findings? | 3 interviews to confirm the results |
| Reporting | | | |
| 29. | Quotations presented | Were participant quotations presented to illustrate the themes / findings? Was each quotation identified? e*.g. participant number* | No, due to limited word count/No |
| 30. | Data and findings consistent | Was there consistency between the data presented and the findings? | Yes |
| 31. | Clarity of major themes | Were major themes clearly presented in the findings? | Yes |
| 32. | Clarity of minor themes | Is there a description of diverse cases or discussion of minor themes? | Yes |
