# Supplementary material for: From Surviving to Living (on): A Grounded Theory Study on Coping in People with Pancreatic Cancer
Source: J Patient Exp. 2023 Nov 20;10:23743735231215605. doi: 10.1177/23743735231215605 (PMC10750439; doi:10.1177/23743735231215605)
Supplement: sj-docx-4-jpx-10.1177_23743735231215605 - Supplemental material for From Surviving to Living (on): A Grounded Theory Study on Coping in People with Pancreatic Cancer [file sj-docx-4-jpx-10.1177_23743735231215605.docx]

Appendix 3: Interview guideline^[[1]](#footnote-1)^

# Interview entry and socio-demographic data^[[2]](#footnote-2)^

*Comparison of the personal data gathered from the short questionnaire collected by telephone^[[3]](#footnote-3)^*

# Phase 1: Explanation phase

*Welcome; introduction; thanks; explanation of the proceedings; explanation of the main points: Coping with illness and support, focus on narrative sequences; questions?*

# Phase 2: General exploration

*Narrative stimuli*

You received your diagnosis a few weeks/months(/years) ago. A lot has certainly happened to you since then. What happened to you as a result of your illness?

*Other questions*

What challenges have you faced due to your illness and how do you deal with them? How do you cope with the consequences, what helps you and what makes it more difficult? Who helps you and in what way?

# Phase 3: Specific exploration

*Reflection (retelling of what has been said and thus the possibility of correcting what has been said)*

*Questions of understanding (ambiguities and vague formulations are addressed)*

*Confrontation (contradictions and unexplained issues are addressed directly in an empathetic way)*

# Phase 4: Ad hoc questions

*Targeting individual topics that have been left out up to this point*

# Closing

*Questions from the participants; thanks; further procedure; farewells*

1. First version. This interview guideline was adapted in the course of the project, see section *data collection*. Translated from German. [↑](#footnote-ref-1)
2. Structure according to Witzel, A. (2000), and Witzel, A. & Reiter, H. (2012). [↑](#footnote-ref-2)
3. Instructions for the interviewer in italics. [↑](#footnote-ref-3)
